# *Rickettsia parkeri* in *Amblyomma americanum* Ticks, Tennessee and Georgia, USA

**DOI:** 10.3201/eid1509.090330

**Published:** 2009-09

**Authors:** Sara B. Cohen, Michael J. Yabsley, Laurel E. Garrison, James D. Freye, Brett G. Dunlap, John R. Dunn, Daniel G. Mead, Timothy F. Jones, Abelardo C. Moncayo

**Affiliations:** Tennessee Department of Health, Nashville, Tennessee, USA (S.B. Cohen, J.R. Dunn, T.F. Jones, A.C. Moncayo); University of Georgia, Athens, Georgia, USA (M.J. Yabsley, D.G. Mead); Georgia Department of Community Health, Atlanta, Georgia, USA (L.E. Garrison); United States Department of Agriculture, Madison, Tennessee, USA (J.D. Freye, B.G. Dunlap)

**Keywords:** Rickettsia parkeri, Amblyomma americanum, Amblyomma maculatum, Rocky Mountain spotted fever, Tennessee, Georgia, bacteria, rickettsia, dispatch

## Abstract

To determine the geographic distribution of the newly recognized human pathogen *Rickettsia parkeri,* we looked for this organism in ticks from Tennessee and Georgia, USA. Using PCR and sequence analysis, we identified *R. parkeri* in 2 *Amblyomma americanum* ticks. This rickettsiosis may be underdiagnosed in the eastern United States.

The most commonly reported rickettsial human pathogen in the United States is *Rickettsia rickettsii,* the causative agent of Rocky Mountain spotted fever (RMSF). First identified in 1937 (*1*), *R. parkeri* has been recognized as a human pathogen only since 2004, when it was isolated from an eschar on a serviceman from Virginia (*2*), although an Ohio patient suspected to have RMSF died of *R. parkeri* rickettsiosis in 1990 (*3*). Little is known about the geographic distribution of *R. parkeri* in the United States or the epidemiology of the disease it causes. The primary vector of *R. parkeri* is thought to be the Gulf Coast tick (*Amblyomma maculatum*), and naturally infected Gulf Coast ticks have been reported in numerous southeastern states (*4*). Experimentally, *A. americanum* ticks can maintain and transmit *R. parkeri* (*5*); thus, this tick species, which is more abundant than *A. maculatum* and bites humans aggressively (*6*), might contribute to *R. parkeri* transmission. To determine the geographic distribution of *R. parkeri*, we examined ticks collected in Tennessee and Georgia.

## The Study

In Georgia, during 2005–2006, residents were encouraged to submit ticks to the state’s Division of Public Health for identification and testing for various tick-borne pathogens. We studied only ticks that had been attached to persons. Ticks were individually homogenized with metal beads and resuspended in 225 μL of phosphate-buffered saline. DNA was extracted from 100 μL of the homogenate by using a QIAamp DNA Micro Kit (QIAGEN Inc, Valencia, CA, USA) according to the manufacturer's instructions. We conducted a nested PCR targeting the 17-kDa gene (*7*) and used bidirectional sequencing at the Integrated Biotechnology Laboratories (University of Georgia, Athens, GA, USA) to confirm positive results.

In Tennessee, from April 2007 through September 2008, the United States Department of Agriculture, Animal and Plant Health Inspection Service, Wildlife Services, and the Tennessee Department of Health collected *A. americanum* and *A. maculatum* ticks from 31 counties by examining wild animals and dragging flannel sheets through vegetation. Ticks were stored in 100% ethanol and were sent to the Tennessee Department of Health Vector-Borne Diseases Laboratory for identification of species and life stage and detection of pathogens by molecular testing. A PCR targeting the outer membrane protein A (*rOmpA*) gene of spotted fever group (SGF) rickettsiae was conducted as previously described, using primers Rr190.602n and Rr190.70p (*8*). To identify the rickettsiae species, we subjected positive samples to a restriction fragment length polymorphism (RFLP) assay by digestion of the amplicons with *Rsa*I (Promega, Madison, WI, USA) and *Pst*I (Fermentas, Glen Burnie, MD, USA) enzymes at 37°C for 2 h. Digested fragments were subjected to electrophoresis on 10% polyacrylamide gels. To confirm species identification, we purified representative positive PCR products with Exosap-It (USB Corporation, Cleveland, OH, USA), sequenced at the Tennessee Department of Health Laboratory Services (Nashville, TN, USA) and entered into the National Center for Biotechnology Information BLAST database (www.blast.ncbi.nlm.nih.gov/Blast.cgi).

Of ticks collected in Georgia, 418 *A. americanum* ticks (237 adults, 180 nymphs, and 1 unknown) and 19 *A. maculatum* ticks, were submitted for testing. Of these, 1 *A. americanum* tick (Fayette County, May 2005) and 1 *A. maculatum* tick (Morgan County, July 2005) were positive for *R. parkeri* (100% identity with GenBank accession no. U17008).

Of 611 *A. americanum* and 2 *A. maculatum* ticks collected in Tennessee, 446 *A. americanum* (164 adults and 282 nymphs) and 2 *A. maculatum* adults were individually tested for *Rickettsia* spp. An additional 103 *A. americanum* larvae were divided in 10 pools of 4–19 ticks each according to collection site. A single *A. americanum* adult male (0.2% of total) had a positive RFLP pattern that matched the previously described *rOmpA* gene pattern of *R. parkeri* (*8*). The sequence of this amplicon (532 bp, GenBank accession no. FJ793521) was 99% similar to that of *R. parkeri* (EU715288). The positive tick had been collected from a coyote in Knox County, Tennessee, in July 2007.

## Conclusions

We identified *R. parkeri* in ticks in Tennessee and Georgia. In Tennessee, our identification of only 2 *A. maculatum* ticks (the primary vector of *R. parkeri*) supports previous reports that this tick species is uncommon in Tennessee (*9*). In Georgia, the 19 *A. maculatum* ticks identified were <5% of the total number of ticks collected in Georgia. In contrast to these low numbers, *A. americanum* ticks are ubiquitous in high densities throughout the other southeastern states and readily feed on humans (*8*). Recently, the range of *A. americanum* ticks has expanded in the United States and now extends from west-central Texas to the Atlantic Coast, encompassing the entire Southeast and parts of the lower Midwest and coastal New England (*10*). The distribution of *A. americanum* ticks completely overlaps the suspected distribution of *A. maculatum* ticks (Figure). The positive *A. americanum* tick from Tennessee was collected from a free-ranging coyote, and the detected *R. parkeri* may have been present in the blood meal taken by the tick. However, *A. americanum* ticks can maintain and transmit *R. parkeri* infection both transovarially and transtadially (*6*). *R. parkeri* has previously been identified in *A. maculatum* ticks from Georgia (*4*), but the identification of *R. parkeri* in *A. americanum* ticks renews concerns that this tick species may be involved in the natural history of another zoonotic pathogen. Additional study is needed to determine the extent of the role of *A. americanum* ticks as a natural vector for *R. parkeri*.

**Figure:**
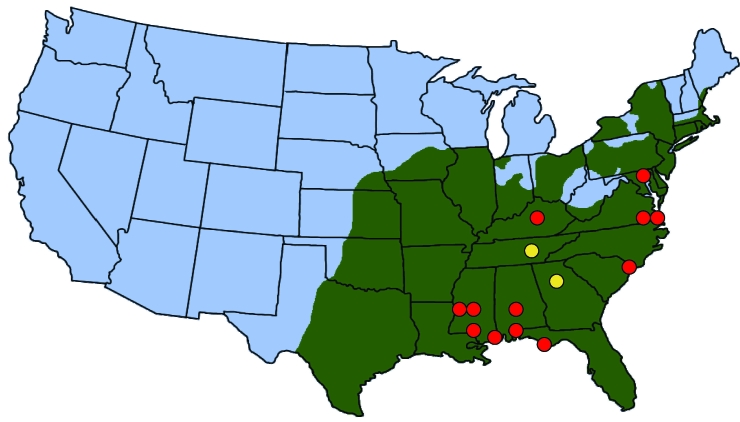
Location of ticks, *Rickettsia parkeri* in ticks, and human cases of rickettsiosis in the United States. Green shading indicates approximate distribution of *Amblyomma americanum* ticks, which completely overlaps with the known or suspected distribution of *A. maculatum.* Yellow circles indicate locations where *R. parkeri* was detected in *A. americanum* ticks (this study). Red circles indicate locations of confirmed or suspected cases of *R. parkeri* infection in humans (*11*).

This study raises concerns about the serologic diagnosis of RMSF. *R. parkeri* may be the etiologic agent of some rickettsiosis cases in Tennessee and Georgia that have been misdiagnosed as RMSF. Because reliable clinical tests specific for different SFG rickettsiae are not readily available, several different rickettsioses may be serologically cross-reactive, leading to misdiagnosis of RMSF (*11*–*14*). Reliable diagnosis requires PCR or culture of biopsy specimens from eschars, when present (*15*). Additional studies characterizing SFG rickettsioses, including development of rickettsial species–specific clinical tests, will assist in attributing rickettsiosis to *R. rickettsii*, *R. parkeri*, or other SFG rickettsial infections.
